# Interleukin‐39 is a novel cytokine associated with type 2 diabetes mellitus and positively correlated with body mass index

**DOI:** 10.1002/edm2.409

**Published:** 2023-02-09

**Authors:** Shahad W. Nussrat, Ali H. Ad'hiah

**Affiliations:** ^1^ Department of Biotechnology, College of Science University of Baghdad Baghdad Iraq; ^2^ Tropical‐Biological Research Unit, College of Science University of Baghdad Baghdad Iraq

**Keywords:** body mass index, Interleukin‐37, Interleukin‐39, type 2 diabetes mellitus

## Abstract

**Introduction:**

It is suggested that cytokines play a key role in the pathogenesis of type 2 diabetes mellitus (T2DM). Therefore, this study explored two recently discovered cytokines, interleukin (IL)‐37 (anti‐inflammatory) and IL‐39 (pro‐inflammatory), in T2DM due to limited data in this context.

**Methods:**

Serum IL‐37 and IL‐39 levels were determined in 106 T2DM patients and 109 controls using enzyme‐linked immunosorbent assay kits.

**Results:**

Serum levels (median and interquartile range) of IL‐37 (79 [47–102] vs. 60 [46–89] ng/L; probability [*p*] = .04) and IL‐39 (66 [59–69] vs. 31 [19–42] ng/L; *p* < .001) were significantly elevated in T2DM patients compared to controls. As indicated by the area under the curve (AUC), IL‐39 (AUC = 0.973; *p* < .001) was more predictable for T2DM than IL‐37 (AUC = 0.582; *p* = .039). Elevated levels of IL‐39 were significantly associated with T2DM (odds ratio = 1.30; *p* < .001), while IL‐37 did not show this association. Classification of IL‐37 and IL‐39 levels by demographic and clinical characteristics of patients revealed some significant differences including gender (IL‐39), body mass index (BMI; IL‐37 and IL‐39) and diabetic neuropathy (IL‐39). BMI was positively correlated with IL‐39 (correlation coefficient [*r*_s_] = 0.27; *p* = .005) and glycosylated haemoglobin (*r*_s_ = 0.31; *p* = .001), and negatively correlated with age at onset (*r*_s_ = −0.24; *p* = .015).

**Conclusions:**

IL‐37 and IL‐39 levels were elevated in the serum of T2DM patients. Besides, IL‐39 is proposed to be a novel cytokine associated with T2DM and positively correlated with BMI.

## Graphical abstract

Serum IL‐37 and IL‐39 levels were up‐regulated in T2DM patients. IL‐39 was an excellent biomarkers in discriminating between T2DM patients and controls. High levels of IL‐39 were significantly associated with T2DM.

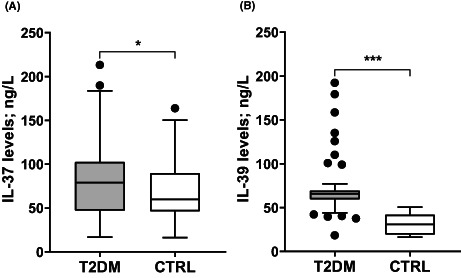

## 1 INTRODUCTION

Type 2 diabetes mellitus (T2DM) is a chronic metabolic disorder, which has attracted healthcare attention and is showing an increasing burden worldwide. The global prevalence of T2DM was estimated at 6000 cases/100,000 population in 2017, and by 2030, it is expected to increase to 7000 cases/100,000 population. In addition, T2DM is associated with more than 1 million deaths annually and ranks ninth among other chronic diseases in causing mortality.^1^ Relative insulin deficiency and resistance, as well as impaired glucose and lipid metabolism, are hallmarks of T2DM. Thus, hyperglycaemia and dyslipidaemia are common features associated with T2DM that increase the risk of long‐term microvascular and macrovascular complications, especially cardiovascular disease and peripheral neuropathy.^2^ The underlying mechanisms involved in the pathophysiology of T2DM are complex and not well defined. However, low‐grade systemic inflammation is a characteristic feature of the disease and elevated systemic levels of inflammatory mediators, such as acute phase proteins and cytokines, are well documented in T2DM patients.^3^ Cytokines are low molecular weight glycoproteins produced by many types of immune and non‐immune cells and secreted into the circulation, where they regulate various aspects of immune responses and inflammatory reactions through their autocrine, paracrine and endocrine actions.^4^ Among them cytokines proposed to be involved in the pathogenesis of T2DM are members of the interleukin (IL)‐1 and IL‐12 cytokine families,^5^, ^6^ but some of these cytokines have been neither extensively explored (i.e., IL‐37) nor investigated (i.e., IL‐39).

IL‐37, a member of the IL‐1 cytokine family identified in 2000, is an anti‐inflammatory cytokine known for its ability to reduce innate inflammation and suppress adaptive immune responses. Many types of immune and non‐immune cells produce IL‐37, including monocytes, macrophages, dendritic cells, B cells, plasma cells, natural killer cells and epithelial cells, in response to pro‐inflammatory stimuli.^7^ Dysregulated production of IL‐37 has been associated with several human inflammatory conditions, such as enterocolitis, Behçet's disease, periodontitis, asthma, SARS‐CoV‐2 infection and liver inflammation.^8^ Regarding T2DM, there is some evidence to suggest that IL‐37 may have a role in the pathogenesis. In this context, IL‐37 has been identified as a key anti‐inflammatory cytokine that has the potential to attenuate obesity‐induced inflammation and insulin resistance.^9^ Besides, up‐regulated expression of IL‐37 has been associated with increased insulin sensitivity in elderly patients with T2DM.^10^ A link between IL‐37 and a dysregulated immune response in COVID‐19 and diabetes has also recently been proposed.^11^

IL‐39, the latest discovered member of the IL‐12 family of cytokines, is heterodimer glycoprotein composed of two covalently linked subunits, IL‐23p19 and Epstein–Barr virus‐induced gene (Ebi3).^12^ In vitro and in vivo studies in mice revealed that IL‐23p19 and Ebi3 are secreted by B cells upon stimulation with lipopolysaccharides (LPS). Dendritic cells and macrophages are other immune cells that also express IL‐23p19 and Ebi3 mRNA, and their expression levels are up‐regulated in the presence of IL‐4 (an anti‐inflammatory cytokine), whereas LPS inhibits this expression in these cells. B cells and plasma cells of lupus‐like mice are also promoted to express high levels of IL‐39.^13^ In humans, there is some controversy about IL‐39 secretion. Ecoeur et al. considered IL‐39 to be a theoretical cytokine and its production and/or biological activity could not be assigned in the human system. The authors speculated that IL‐39 might be an immunoregulatory cytokine only in mice.^14^ On the contrary, detectable levels of IL‐39 have been demonstrated in human serum.^15^, ^16^ Research on the immune functions of IL‐39 is still in its preliminary stage. However, pro‐inflammatory effects of IL‐39 have been suggested in lupus‐like mice, and thus this cytokine may contribute to the immunopathogenic mechanisms involved in systemic lupus erythematosus (SLE).^12^ Further, significantly elevated levels of IL‐39 were found in the serum of patients with acute coronary syndrome and the authors suggested that IL‐39 could be considered an indicator of systolic dysfunction.^15^ In patients with neuromyelitis optica spectrum disorders, an inflammatory demyelinating autoimmune disorder, up‐regulated levels of IL‐39 have been demonstrated.^16^ IL‐39 has also been associated with exacerbation of concanavalin A‐induced hepatitis.^17^ These studies point to the potential involvement of IL‐39 in the pathogenesis of human inflammatory diseases. Since low‐grade inflammation is a common feature of T2DM,^3^ IL‐39 may be a relevant cytokine whose pathogenic role in disease should be understood.

This study planned with the aims to evaluate IL‐37 and IL‐39 levels in the serum of patients with T2DM in order to assess their biomarker significance and association with disease. In addition, the correlation between IL‐37 and IL‐39 levels and demographic and clinical characteristics of T2DM was also evaluated.

## 2 MATERIALS AND METHODS

### 2.1 Subjects and study design

A study was conducted on 106 patients diagnosed with T2DM (mean age = 54 ± 10 years, minimum age = 34 years, maximum age = 75 years, 52 [49.1%] males, 54 [50.9%] females). Patients were referred to the National Diabetes Center (Mustansiriyah University) in Baghdad during 15 November 2021—10 February 2022. Diagnosis was based on assessment of fasting plasma glucose (FPG ≥126 mg/dL) and glycosylated haemoglobin (HbA1c ≥ 6.5%) according to the diagnostic criteria established by the American Diabetes Association (ADA).^18^ The modified Toronto Clinical Neuropathy Scoring (TCNS) system was followed to assess diabetic sensorimotor polyneuropathy in patients.^19^ Inclusion criteria were adults (18 years and older), non‐insulin dependent T2DM and adult onset diabetes. Exclusion criteria were type 1 DM, gestational diabetes, diabetes complications other than neuropathy, chronic diseases, cardiovascular complications and cancer. The control sample (CTRL) included 109 age‐ and gender‐matched healthy individuals (mean age = 52 ± 8 years, minimum age = 36 years, maximum age = 71 years, 54 [49.5%] males, 55 [50.5%] females). They were blood donors and health service personnel who did not have signs or symptoms of diabetes and random blood glucose (80 ± 6 mg/dL) and HbA1c (5.4 ± 0.4%) were within the reference range. Written consent was obtained from all participants and the study protocol was approved by the Ethics Committee of the College of Science, University of Baghdad (approval reference: CSEC/0122/0021).

### 2.2 Anthropometric and biochemical measurements

Anthropometric measurement included body mass index (BMI), which was calculated by dividing weight (kilogram; kg) by the square of height (meter; m). According to the scheme of the World Health Organization (WHO), T2DM patients were classified into three categories of BMI; normal‐weight (18.5–24.9 kg/m^2^), overweight (25–29.9 kg/m^2^) and obese (≥ 30 kg/m^2^).^20^ Biochemical measurements included the following parameters: FPG, HbA1c, alanine aminotransferase (ALT), aspartate aminotransferase (AST), alkaline phosphatase (ALP), total cholesterol, triglycerides, high‐density lipoprotein (HDL) and low‐density lipoprotein (LDL). An automated biochemical analyser (Cobas c 311analyzer, Cobas‐Roche, Germany) pre‐loaded with respective reagents was used to measure the biochemical parameters. A blood sample (5 mL) was collected in a plain tube after 8–10 hours of fasting, and after coagulation for at least 30 minutes at room temperature (20–25°C), the tube was centrifuged (1000 *× g*, 15 min). Separated serum was frozen at −20°C until assessment.

### 2.3 IL‐37 and IL‐39 immunoassay

Enzyme‐linked immunosorbent assay (ELISA) kits were used to measure serum levels of IL‐37 (Catalogue Number: MBS165041) and IL‐39 (Catalogue Number: MBS167915), following the manufacturer's instructions (MyBioSource, Inc., USA). Both kits were based on similar principles, sandwich ELISA. The 96‐well plate was pre‐coated with anti‐human IL‐37 or IL‐39 antibody. Standards and serum samples were added to pre‐assigned wells, and after a period of incubation, the wells were washed. This was followed by the addition of a biotinylated anti‐human IL‐37 or IL‐39 antibody. After incubation and washing, streptavidin‐horseradish peroxidase (HRP) was added. After an incubation period, each well was washed to remove unbound streptavidin‐HRP. Then, substrate solution was added and the reaction was terminated by adding stop solution. The absorbance of each well was measured at a wavelength of 450 nm using an ELISA reader (HumaReader HS, HUMAN Gesellschaft für Biochemica und Diagnostica mbH, Germany). The cytokine concentration of unknown samples was measured by plotting a standard curve and generating a curve‐fitting equation. The standard curve range of IL‐37 and IL‐39 was 0–480 ng/L and 0–640 ng/L respectively.

### 2.4 Statistical analysis

Categorical variables were given as number and frequency (percentage). Significant differences between frequencies were evaluated using Pearson Chi‐square test. Normal distribution of continuous variables was tested using the Kolmogorov–Smirnov and Shapiro–Wilk normality tests. Normally distributed (parametric) variables were given as mean and standard deviation (SD). Significant differences between means were assessed using one‐way analysis of variance (ANOVA) test. Non‐parametric variables, which did not show a normal distribution, were expressed as median and interquartile range (IQR; 25%–75%). Significant differences between medians were determined using the Mann–Whitney U test (for comparison of two groups) or Kruskal–Wallis test (for comparison of more than two groups). Receiver operating characteristic (ROC) curve analysis was performed to evaluate the diagnostic performance of IL‐37 and IL‐39 in T2DM, according to the area under the curve (AUC), cut‐off value, sensitivity and specificity. The cut‐off value was optimized using Youden index (YI) of the ROC curve. Multinomial logistic regression analysis was performed to calculate odds ratio (OR) and its 95% confidence interval (CI). Correlation of IL‐37 and IL‐39 with some clinical and laboratory features of T2DM was analysed using two‐tailed Spearman's rank‐order correlation. The calculated correlation coefficient (*r*_s_) was presented as a heat‐map matrix. A probability value (*p*) ≤ .05 was considered statistically significant. IBM SPSS Statistics 25.0 (Armonk, NY: IBM Corp) and GraphPad Prism version 8.0.0 (San Diego, CA, USA) were used to perform statistical analysis. G*power software (version 3.1.9.7) was used to determine the power of the sample size.^21^

## 3 RESULTS

### 3.1 Power of sample size

The power of the sample size was determined using the G*power software with the following input parameters: number of T2DM patients = 106; number of CTRL = 109; two‐tailed α‐error *p* = .05; effect size d = 0.5. The estimated power of sample size (1‐β error *p*) was .95. The statistically acceptable power for the sample size is 0.8.^22^

### 3.2 Baseline demographic and clinical characteristics

Baseline demographic and clinical characteristics are given in Table 1. The mean age of T2DM patients was higher than that of CTRL but the difference was not significant (54 ± 10 vs. 52 ± 8; *p* = .272). Similarly, patients and CTRL classified by age group (34–45, 46–60, and > 60 years) or gender (male and female) also showed no significant differences (*p* = .963 and 0.943 respectively). The patients' BMI was 32.0 ± 5.9 kg/m^2^, which was above the normal weight range (18.5–24.9 kg/m^2^). In fact, most patients with T2DM were either overweight (34.9%) or obese (56.6%) (Overweight/obese = 91.5%). Further, 90.6% of patients had a family history of DM. Diabetic neuropathy was found in 19.8% of patients and it was mostly mild neuropathy (TCNS score = 6–8). The mean age at onset of T2DM was 46 ± 8 years with disease duration mean of 9.5 ± 5.6 years.

**TABLE 1:** Baseline characteristics of type 2 diabetes mellitus patients and controls.

Characteristic; mean ± SD or n (%)		T2DM; *n* = 106	CTRL; *n* = 109	*p*‐value	Reference range
Age; years		54 ± 10	52 ± 8	.272	NA
Age group; years	34–45	22 (20.7)	23 (21.1)	.963	NA
	46–60	59 (55.7)	62 (56.9)		
	> 60	25 (23.6)	24 (22.0)		
Gender	Male	52 (49.1)	54 (49.5)	.943	NA
	Female	54 (50.9)	55 (50.5)		
Body mass index; kg/m^2^		32.0 ± 5.9	NA		18.5–24.9
Body mass index group	Normal‐weight	9 (8.5)	NA		NA
	Overweight	37 (34.9)			
	Obese	60 (56.6)			
Family history	Yes	96 (90.6)	NA		NA
	No	10 (9.4)			
Diabetic neuropathy	Yes	21 (19.8)	NA		NA
	No	85 (80.2)			
Age at onset; years		46 ± 8	NA		NA
Disease duration; years		9.5 ± 5.6	NA		NA
Disease duration group; years	≤ 1	11 (10.4)	NA		NA
	2–5	34 (32.1)			
	6–10	37 (34.9)			
	> 10	24 (22.6)			
FPG; mg/dL		198 ± 78	80 ± 6	**<.001**	70–100
FPG percentile (mg/dL)	< 25 (80–128)	26 (24.5)	NA		NA
	25–49 (129–179)	24 (22.6)			
	50–74 (180–250)	29 (27.4)			
	≥ 75 (254–397)	27 (25.5)			
HbA1c; %		8.8 ± 2.0	5.4 ± 0.4	**<.001**	≤5
HbA1c percentile (%)	< 25 (4.7–7.5)	27 (25.5)	NA		NA
	25–49 (7.6–8.3)	23 (21.7)			
	50–74 (8.4–10.0)	28 (26.4)			
	≥ 75 (10.1–15.0)	28 (26.4)			
ALT; U/L		21 ± 27	NA		10–35
AST; U/L		28 ± 22	NA		0–35
ALP; IU/L		115 ± 76	NA		42–136
Total cholesterol; mg/dL		190 ± 53	NA		<200
Triglycerides; mg/dL		146 ± 68	NA		<150
HDL; mg/dL		45 ± 30	NA		>50
LDL; mg/dL		116 ± 52	NA		<100

Abbreviations: ALP, Alkaline phosphatase; ALT, Alanine aminotransferase; AST, Aspartate aminotransferase; CTRL, Healthy controls; FPG, Fasting plasma glucose; HbA1c, Glycosylated haemoglobin; HDL, High‐density lipoprotein; LDL, Low‐density lipoprotein; NA, Not applicable; *p*, Probability of one‐way analysis of variance test (to compare continuous variables) or Pearson Chi‐square test (to compare categorical variables); T2DM, Type 2 diabetes mellitus; SD, Standard deviation.

### 3.3 Baseline laboratory data

FPG (198 ± 78 vs. 80 ± 6 mg/dL; *p* < .001) and HbA1c (8.8 ± 2.0 vs. 5.4 ± 0.4%; *p* < .001) were significantly elevated in T2DM patients compared with CTRL. In addition, both parameters (FPG and HbA1c) in patients were above the reference ranges set by the ADA (FPG ≥126 mg/dL; HbA1c ≥ 6.5%).^18^ Patients classified into percentiles (< 25, 25–49, 50–74, and ≥ 75%) according FPG or HbA1c levels showed similar frequencies in each classification (FPG percentiles: 24.5, 22.6, 27.4 and 25.5% respectively; HbA1c percentiles: 25.5, 21.7, 26.4 and 26.4%) respectively. ALT (21 ± 27 U/L), AST (28 ± 22 U/L), ALP (115 ± 76 IU/L) and HDL (45 ± 30 mg/dL) concentrations were within the reference ranges, while the concentrations of total cholesterol (190 ± 53 vs. < 200 mg/dL) and triglycerides (146 ± 68 vs. < 150 mg/dL) were close to the upper reference limits and LDL concentration was above the reference range (116 ± 52 vs. <100 mg/dL) (Table 1).

### 3.4 IL‐37 and IL‐39 levels

**FIGURE 1:**
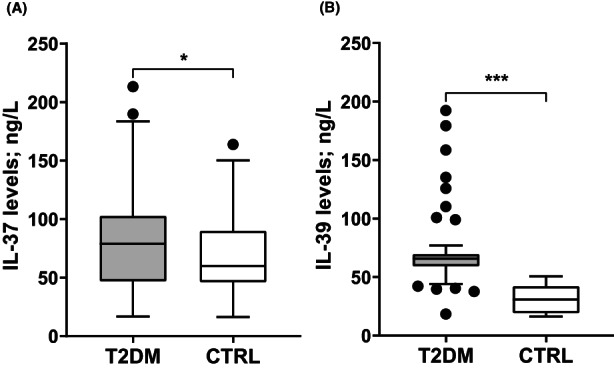
Box and whisker plot (Tukey method) of serum interleukin (IL)‐37 (A) and IL‐39 (B) levels in type 2 diabetes mellitus (T2DM) patients and controls (CTRL). Horizontal line inside the box indicates median. Whiskers indicate the interquartile range (IQR). Black circles indicate the outliers. IL‐37 (79 [IQR: 47–102] vs. 60 [IQR: 46–89] ng/L; *p* = .04) and IL‐39 (66 [IQR: 59–69] vs. 31 [IQR: 19–42] ng/L; *p* < .001) levels were significantly elevated in patients compared to CTRL. Significance was assessed using the Mann–Whitney U test (*p < .05; ***p < .001).

**FIGURE 2:**
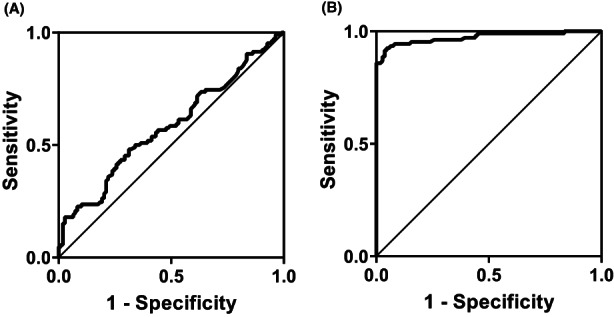
Receiver operating characteristic (ROC) curve analysis of interleukin (IL)‐37 (A) and IL‐39 (B) in type 2 diabetes mellitus (T2DM). As indicated by ROC curve analysis, IL‐39 (area under the curve [AUC] = 0.973; 95% confidence interval [CI] = 0.951–0.995; cut‐off value = 46 ng/L; Youden index [YI] = 0.87; sensitivity = 93.4%; specificity = 93.6%; *p* < .001) was more predictable for T2DM than IL‐37 (AUC = 0.582; 95% CI = 0.505–0.658; cut‐off value = 69 ng/L; YI = 0.87; sensitivity = 56.6%; specificity = 56.0%; *p* = .039).

Normality tests revealed that serum levels of IL‐37 and IL‐39 did not follow a normal distribution, therefore they were presented as median and IQR. The levels of IL‐37 (79 [IQR: 47–102] vs. 60 [IQR: 46–89] ng/L; *p* = .04) and IL‐39 (66 [IQR: 59–69] vs. 31 [IQR: 19–42] ng/L; *p* < .001) were significantly elevated in T2DM patients compared to CTRL (Figure 1). As indicated by the AUC, ROC curve analysis demonstrated that IL‐39 (AUC = 0.973; 95% CI = 0.951–0.995; cut‐off value = 46 ng/L; YI = 0.87; sensitivity = 93.4%; specificity = 93.6%; *p* < .001) was more predictable for T2DM than IL‐37 (AUC = 0.582; 95% CI = 0.505–0.658; cut‐off value = 69 ng/L; YI = 0.87; sensitivity = 56.6%; specificity = 56.0%; *p* = .039) (Figure 2). Multinomial logistic regression analysis (CTRL was the reference category) showed that elevated levels of IL‐39 were significantly associated with T2DM (OR = 1.30; 95% CI = 1.19–1.42; *p* < .001), while IL‐37 did not show this association (OR = 1.01; 95% CI = 0.99–1.02; *p* = .558) (Table 2).

**TABLE 2:** Multinomial logistic regression analysis of IL‐37 and IL‐39 in type 2 diabetes mellitus (controls were the reference category).

Cytokine	B	SE	Wald	DF	*p*‐value	Exp(B)	95% CI
IL‐37	0.01	0.01	0.34	1	.558	1.01	0.99–1.02
IL‐39	0.26	0.04	34.39	1	**<.001**	1.30	1.19–1.42

*Note*: *p*: Two‐tailed probability (significant *p*‐value is indicated by bold).

Abbreviations: B, Multinomial logistic regression coefficient; CI, Confidence interval; DF, Degrees of freedom; Exp(B), Odds ratio for the predictors; IL, Interleukin; SE, Standard error.

### 3.5 Classification of IL‐37 and IL‐39 levels by patient's characteristics

When IL‐37 and IL‐39 were classified according to age group, gender, BMI, family history, diabetic neuropathy, disease duration and FPG percentiles, only four significant differences were found. IL‐39 levels were significantly elevated in females compared to males (68 [IQR: 63–71] vs. 64 [IQR: 57–68] ng/L; *p* = .01). Serum IL‐37 and IL‐39 levels were significantly different between BMI groups (*p* = .017 and .021 respectively). Serum IL‐39 levels were significantly decreased in patients with diabetic neuropathy compared with those without neuropathy (64 [IQR: 47–68] vs. 67 [IQR: 61–70] ng/L; *p* = .022) (Table 3).

**TABLE 3:** IL‐37 and IL‐39 levels (median and interquartile range) stratified by characteristics of type 2 diabetes mellitus patients.

Characteristic	IL‐37; ng/L	*p*‐value	IL‐39; ng/L	*p*‐value
Age; years				
34–45	81 (46–119)	.848	68 (64–70)	.319
46–60	80 (46–99)		66 (59–69)	
> 60	70 (49–112)		65 (60–68)	
Gender				
Male	71 (47–98)	.316	64 (57–68)	**.01**
Female	81 (49–116)		68 (63–71)	
Body mass index				
Normal‐weight	129 (98–137)	**.017**	65 (51–70)	**.021**
Overweight	55 (47–96)		62 (57–67)	
Obese	79 (46–99)		67 (63–71)	
Family history				
Yes	81 (49–108)	.06	65 (59–70)	.634
No	48 (43–80)		67 (60–68)	
Diabetic neuropathy				
Yes	82 (45–101)	.754	64 (47–68)	**.022**
No	76 (48–102)		67 (61–70)	
Disease duration; years				
≤ 1	80 (47–126)	.474	67 (61–72)	.66
2–5	88 (49–127)		67 (61–70)	
6–10	69 (45–100)		65 (58–68)	
> 10	72 (51–94)		64 (58–69)	
FPG percentile (mg/dL)				
< 25 (80–128)	60 (46–115)	.097	65 (60–69)	.387
25–49 (129–179)	87 (51–123)		64 (57–70)	
50–74 (180–250)	54 (44–91)		66 (58–68)	
≥ 75 (254–397)	86 (61–100)		68 (59–71)	

*Note*: *p*: two‐tailed probability (significant *p*‐value is indicated by bold). Statistically significant differences between medians were evaluated using the Mann–Whitney *U*‐test (to compare two groups) or Kruskal–Wallis test (to compare more than two groups).

Abbreviation: FPG, Fasting plasma glucose.

### 3.6 Spearman's rank correlation analysis

**FIGURE 3:**
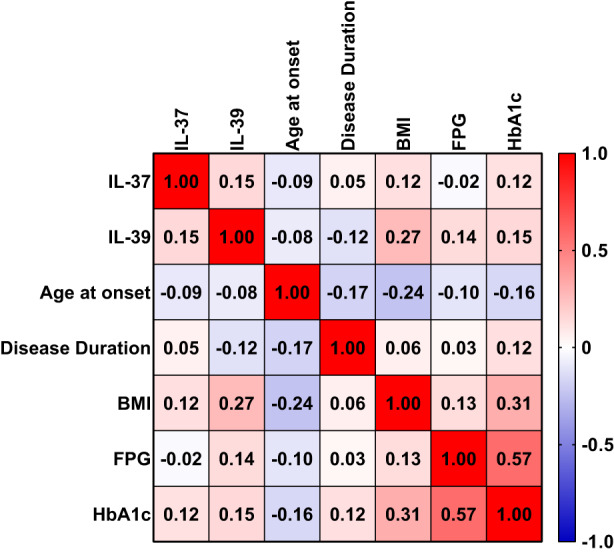
A heat‐map matrix of correlation of IL‐37 and IL‐39 with age at disease onset, disease duration, body mass index (BMI), fasting plasma glucose (FPG) and glycosylated haemoglobin (HbA1c) in type 2 diabetes mellitus patients. The values inside the squares indicate the Spearman rank correlation coefficient (*r*_s_). Four significant correlations were found. BMI was positively correlated with IL‐39 (*r*_s_ = 0.27; *p* = .005) and HbA1c (*r*_s_ = 0.31; *p* = .001), and negatively correlated with age at onset (*r*_s_ = −0.24; *p* = .015). FPG was positively correlated with HbA1c (*r*_s_ = 0.57; *p* < .001).

Spearman's rank correlation analysis of IL‐37 and IL‐39 in relation to some demographic and clinical characteristics of T2DM patients (age at onset, disease duration, BMI, FPG and HbA1c) was performed and presented as a heat‐map (Figure 3). Four significant correlations were found. BMI was positively correlated with IL‐39 (*r*_s_ = 0.27; *p* = .005) and HbA1c (r_s_ = 0.31; *p* = .001), and negatively correlated with age at onset (*r*_s_ = −0.24; *p* = .015). FPG was positively correlated with HbA1c (*r*_s_ = 0.57; *p* < .001).

## 4 DISCUSSION

The current study examined serum levels of IL‐37 and IL‐39 in patients with T2DM and age‐ and gender‐matched CTRL. The diagnosis of T2DM was confirmed, with most patients showing levels of FPG and HbA1c exceeding the thresholds established by the ADA criteria. These criteria recommend that FPG of 126 mg/dL or higher and HbA1c of 6.5% or higher be diagnostic thresholds for T2DM.^18^ However, we noted that some patients showed normal levels for both tests (about 25%). This was probably due to the use of oral hypoglycaemic drugs, and indicated that these patients had good control of hyperglycaemia, while the others had poor control of hyperglycaemia, even though they were taking oral hypoglycaemic agents. Since the glycaemia status may have an effect on the production of cytokines,^23^ the levels of FPG and HbA1c were classified into percentiles to reach a better understanding of IL‐37 and IL‐39 role in the pathogenesis of T2DM. However, no significant differences were found and IL‐37 and IL‐39 levels appeared to be unaffected by FPG and HbA1c.

Most of T2DM patients were either overweight or obese (91.5%), and this may link obesity to T2DM aetiology. Consistent with this observation, studies have considered obesity as the most significant risk factor associated with T2DM and weight loss by 5% has been associated with a good improvement of glycaemia control.^24^ Furthermore, epidemiological studies indicated that the global increase in obesity prevalence paralleled the increase in T2DM prevalence in a concomitant manner.^25^ Besides, circulating cytokine levels have been shown to be influenced by obesity.^26^ Therefore, this issue was addressed in the current study in relation to IL‐37 and IL‐39. The study also addressed the issue of diabetic neuropathy, one of the most important complications associated with T2DM, as about 50% of T2DM patients eventually develop diabetic neuropathy.^27^ In addition, a recent study demonstrated that inflammatory cytokines, particularly tumour necrosis factor (TNF)‐α, are important predictors of diabetic neuropathy over a period of 5 years.^28^ In the current study, about 20% of T2DM patients suffered from diabetic neuropathy, and probably the frequency was higher if we included a larger number of patients. The study also indicated that 90.6% of T2DM patients had a family history of disease (siblings, parents or grandparents). It is generally agreed that T2DM shows a tendency to increase in families and individuals with affected parents and/or sibs are at a greater risk to develop T2DM. This also highlight the importance of genetic predisposition to develop T2DM.^29^, ^30^

The main concern of the current study was to understand the significance of IL‐37 and IL‐39 as biomarkers of T2DM, as available data in this regard are limited. The results pointed out for their significance in the pathogenesis of T2DM, particularly IL‐39. Both cytokines showed significantly higher levels in the serum of T2DM patients compared to CTRL. ROC curve analysis demonstrated that IL‐39 was excellent in differentiating between T2DM and CTRL (AUC = 0.973). Based on these findings, IL‐39 can be considered as a novel predictor of T2DM and/or a therapy target in the disease. For IL‐37, although serum levels were significantly higher in patients, the ROC curve and multinomial regression analyses did not indicate the usefulness of IL‐37 as a biomarker of T2DM.

IL‐37 is a novel cytokine of the IL‐1 family that have shown up‐regulated serum levels in T2DM patients, but ROC curve analysis revealed no predictive value (AUC = 0.582) and multinomial logistic regression analysis did not support an association with the disease risk (OR = 1.01; *p* = .558). This cytokine is known to have anti‐inflammatory functions and during inflammation, IL‐37 has been found to show an increased expression to counteract and prevent excessive inflammatory reactions.^9^ Therefore, the up‐regulated serum levels of IL‐37 may be due to a feedback mechanism to counter pro‐inflammatory cytokines that exhibit elevated serum levels in T2DM.^3^ The current study results were in agreement with those of a recent study, which indicated that serum IL‐37 and mRNA levels were up‐regulated in elderly patients with T2DM (aged 65–95 years).^10^ In the current study, patients over 60 years of age also showed elevated levels of IL‐37 compared to corresponding CTRL (70 [IQR: 49–112] vs. 64 [IQR: 47–86] ng/L), but the difference was only marginally significant (*p* = .054). Another study examined IL‐37 expression in kidney cells (podocytes) of T2DM patients with diabetic nephropathy, an important complication associated with diabetes. It was found that IL‐37 was significantly associated with a decrease in hyperglycemia‐induced podocyte inflammation, indicating the protective effects of IL‐37 against podocyte injury. In addition, IL‐37 was significantly associated with a decrease in oxidative stress and apoptosis induced by hyperglycaemia. Moreover, STAT3 and cyclophilin A, two proteins involved in pro‐inflammatory and apoptotic signalling pathways, can be inhibited by IL‐37.^31^ T2DM patients with neuropathy in this study also showed elevated serum levels of IL‐37 compared to patients without neuropathy (82 vs. 76 ng/L) but the difference was no significant. The IL‐37 role in T2DM pathogenesis has also been explored in animal models and there has been evidence demonstrating the anti‐inflammatory effects of IL‐37. The use of recombinant IL‐37 for treatment in mice with metabolic syndrome was associated with improved insulin sensitivity and lower levels of pro‐inflammatory cytokines.^32^

IL‐39, the next cytokine examined, has shown an association with T2DM (OR = 1.30) and its usefulness as a novel biomarker for the disease has been proposed (AUC = 0.973). It was also interesting to note that IL‐39 was positively correlated with BMI. Unfortunately, this cytokine has not been studied in T2DM, and the current study may be the first to examine serum IL‐39 levels in patients with T2DM. However, evidence has been presented to highlight a role for IL‐39 in the risk of other diseases. Since the discovery in 2016, the pro‐inflammatory effects of IL‐39 have been identified and causally linked to SLE in lupus‐prone mice.^33^ In patients with acute coronary syndrome, serum levels of IL‐39 were also significantly increased and showed a positive correlation with high‐sensitivity C‐reactive protein (CRP). Its potential has also been suggested as an indicator of cardiac systolic dysfunction.^15^ Moreover, IL‐39 levels were significantly up‐regulated in the serum of patients with neuromyelitis optica spectrum disorders compared with CTRL, and were positively correlated with disease severity.^16^ Another study indicated that IL‐39 was associated with the exacerbation of concanavalin A‐induced liver inflammation, and based on this, IL‐39 was proposed as a therapeutic target in inflammatory hepatic diseases.^17^ Besides, the pathogenic role of IL‐39 in acute graft‐versus‐host disease has been identified and its role in potentiating T‐lymphocyte functions has been recognized.^34^ However, contradictory results have been reported in patients with Graves' disease and Hashimoto's thyroiditis, and IL‐39 showed down‐regulated levels but was positively correlated with two inflammatory markers, CRP and leukocyte count.^35^ Based on these findings and as shown in the results of this study, it may be reasonable to suggest that IL‐39 is also a T2DM‐associated cytokine and could be considered as a potential biomarker for the disease.

When IL‐39 levels were examined in subgroups of T2DM patients, two significant differences were found. Female patients showed elevated levels compared to male patients, and patients with neuropathy showed low levels compared to patients without neuropathy. Although no data are available on sex‐related differences in IL‐39 levels, experimental in vivo evidence has indicated that inflammatory responses differ between males and females. It has been shown that inflammatory responses to bacterial endotoxins were higher in women than in men, with significantly higher plasma levels of the pro‐inflammatory cytokines TNF‐α and IL‐6 and the steroid hormone cortisol.^36^ Besides, as in T2DM, there is evidence that females develop low‐grade generalized inflammation and elevated levels of inflammatory markers, such as pro‐inflammatory cytokines and CRP, during the onset of menstruation when oestrogen levels are lowest. Therefore, inflammation and immunity in women are complicated by sex hormones, especially oestrogen, as well as menopause because pre‐menopausal women have higher levels of oestrogen than post‐menopausal women.^37^ Unfortunately, we did not measure serum levels of oestrogen and other sex hormones in current T2DM patients, and this issue warrants exploration and may aid in a better understanding of IL‐39 role in T2DM. With regard to peripheral neuropathy, surprisingly, IL‐39 levels were significantly lower in patients with neuropathy than in patients without neuropathy. Because, a recent five‐year prospective cohort study indicated that elevated levels of pro‐inflammatory cytokines are good predictors of diabetic neuropathy.^28^ There is no clear explanation for this difference but the number of patients with diabetic neuropathy (*n* = 21) may have an effect. In addition, only patients with mild neuropathy were included. Therefore, this issue needs to be explored further by including more patients and covering the full range of TCNS score.

It was interesting to note that IL‐39 levels were positively correlated with BMI. BMI may indirectly indicate obesity and the current T2DM patients were either obese or overweight (91.5%). In fact, studies have identified obesity as a complex disease associated with elevated levels of inflammatory markers, which can lead to a chronic, low‐grade inflammation with effects on the functions of immune cells, such as macrophages.^24^, ^25^, ^38^ Researchers found that adipose tissue in obese subjects was linked to a shift of anti‐inflammatory M2 macrophages to pro‐inflammatory M1 macrophages, and this was associated with insulin resistance. In this context, the adipose tissue of obese subjects has been found to secrete more pro‐inflammatory markers that parallel the development of T2DM.^39^ Based on these observations and because of the pro‐inflammatory function of IL‐39, the positive correlation between this cytokine and BMI in T2DM patients could be explained, but further studies are needed to confirm or refute this explanation.

The study faced several limitations. First, although the sample size has been validated statistically, it is still relatively small especially when patients are stratified into subgroups based on certain demographic, anthropological and clinical characteristics, such as age group, gender, BMI and diabetic neuropathy. Second, homeostatic model assessment (HOMA) was not performed and this may shed light on the relationship between IL‐39 levels, β‐cell function and insulin resistance. Third, the results were not evaluated in light of the type of hypoglycaemic medications. Fourth, the study did not examine sex hormones and other cytokines of the IL‐1 and IL‐12 families as this may expand our understanding of the relationship between these analytes and low‐grade inflammation in T2DM patients. Equally important, a more informative profile can be obtained in this context if pre‐diabetic subjects, as well as newly diagnosed cases, are included.

In conclusion, IL‐37 and IL‐39 levels were elevated in the serum of T2DM patients. Besides, IL‐39 is proposed to be a novel cytokine associated with T2DM and positively correlated with BMI.

## AUTHOR CONTRIBUTIONS

Shahad Nussrat: Conceptualization, Visualization, Data curation, Methodology, Investigation, Validation, Writing–Reviewing and Editing. Ali Ad'hiah: Conceptualization, Visualization, Methodology, Investigation, Supervision, Software, Validation, Writing–Reviewing and Editing.

## FUNDING INFORMATION

No funds have been received.

## CONFLICT OF INTEREST STATEMENT

The authors declare that they have no potential financial or non‐financial conflicts of interest.

## Data Availability

The data sets used and/or analysed during the current study are available from the corresponding author upon reasonable request.

## References

[1] Khan MAB , Hashim MJ , King JK , Govender RD , Mustafa H , Al KJ . Epidemiology of type 2 diabetes—global burden of disease and forecasted trends. J Epidemiol Glob Health. 2020;10(1):107‐111. doi:10.2991/JEGH.K.191028.001 32175717 PMC7310804

[2] Galicia‐Garcia U , Benito‐Vicente A , Jebari S , et al. Pathophysiology of type 2 diabetes mellitus. Int J Mol Sci. 2020;21(17):1‐34. doi:10.3390/ijms21176275 PMC7503727 32872570

[3] Velikova TV , Kabakchieva PP , Assyov YS , Georgiev TA . Targeting inflammatory cytokines to improve type 2 diabetes control. Biomed Res Int. 2021;2021:7297419‐7297412. doi:10.1155/2021/7297419 34557550 PMC8455209

[4] Turner MD , Nedjai B , Hurst T , Pennington DJ . Cytokines and chemokines: At the crossroads of cell signalling and inflammatory disease. Biochim Biophys Acta—Mol Cell Res. 2014;1843(11):2563‐2582. doi:10.1016/j.bbamcr.2014.05.014 24892271

[5] Banerjee M , Saxena M . Interleukin‐1 (IL‐1) family of cytokines: role in type 2 diabetes. Clin Chim Acta. 2012;413(15–16):1163‐1170. doi:10.1016/j.cca.2012.03.021 22521751

[6] Nam H , Ferguson BS , Stephens JM , Morrison RF . Impact of obesity on IL‐12 family gene expression in insulin responsive tissues. Biochim Biophys Acta—Mol Basis Dis. 2013;1832(1):11‐19. doi:10.1016/j.bbadis.2012.08.011 PMC3545633 22952004

[7] Wang M . The role of IL‐37 and IL‐38 in obstetrics abnormalities. Front Med. 2021;8:737084. doi:10.3389/fmed.2021.737084 PMC8429600 34513891

[8] Su Z , Tao X . Current understanding of IL‐37 in human health and disease. Front Immunol. 2021;12:12. doi:10.3389/fimmu.2021.696605 PMC8267878 34248996

[9] Ballak DB , Van Diepen JA , Moschen AR , et al. IL‐37 protects against obesity‐induced inflammation and insulin resistance. Nat Commun. 2014;5:5. doi:10.1038/ncomms5711 25182023

[10] Li T , Li H , Li W , et al. Interleukin‐37 sensitize the elderly type 2 diabetic patients to insulin therapy through suppressing the gut microbiota dysbiosis. Mol Immunol. 2019;112:322‐329. doi:10.1016/j.molimm.2019.06.008 31238287

[11] Tokajian S , Merhi G , Al Khoury C , Nemer G . Interleukin‐37: a link between COVID‐19, diabetes, and the black fungus. Front Microbiol. 2022;12:4328. doi:10.3389/fmicb.2021.788741 PMC8793130 35095801

[12] Wang X , Wei Y , Xiao H , et al. A novel IL‐23p19/Ebi3 (IL‐39) cytokine mediates inflammation in lupus‐like mice. Eur J Immunol. 2016;46(6):1343‐1350. doi:10.1002/eji.201546095 27019190 PMC11334612

[13] Lu Z , Xu K , Wang X , Li Y , Li M . Interleukin 39: a new member of interleukin 12 family. Cent Eur J Immunol. 2020;45(2):214‐217. doi:10.5114/ceji.2020.97911 33456334 PMC7792434

[14] Ecoeur F , Weiss J , Schleeger S , Guntermann C . Lack of evidence for expression and function of IL‐39 in human immune cells. PLoS One. 2020;15(12):e0242329. doi:10.1371/journal.pone.0242329 33259477 PMC7707563

[15] Luo Y , Liu F , Liu H , et al. Elevated serum IL‐39 in patients with ST‐segment elevation myocardial infarction was related with left ventricular systolic dysfunction. Biomark Med. 2017;11(6):419‐426. doi:10.2217/bmm-2016-0361 28379039

[16] Yang MG , Tian S , Zhang Q , et al. Elevated serum interleukin‐39 levels in patients with neuromyelitis optica spectrum disorders correlated with disease severity. Mult Scler Relat Disord. 2020;46:102430. doi:10.1016/j.msard.2020.102430 32853892

[17] Li Y , Gong L , Weng L , Pan X , Liu C , Li M . Interleukin‐39 exacerbates concanavalin A‐induced liver injury. Immunopharmacol Immunotoxicol. 2021;43(1):94‐99. doi:10.1080/08923973.2020.1869778 33412981

[18] American‐Diabetes‐Association . Classification and diagnosis of diabetes: standards of medical Care in Diabetes‐2020. Diabetes Care. 2020;43(Suppl 1):S14‐S31. doi:10.2337/dc20-S002 31862745

[19] Bril V , Tomioka S , Buchanan RA , Perkins BA . Reliability and validity of the modified Toronto clinical neuropathy score in diabetic sensorimotor polyneuropathy. Diabet Med. 2009;26(3):240‐246. doi:10.1111/j.1464-5491.2009.02667.x 19317818 PMC2871179

[20] Weir CB , Jan A . BMI classification percentile and cut off points. StatPearls Publishing; 2019 https://www.ncbi.nlm.nih.gov/books/NBK541070/. Accessed March 16, 2022. 31082114

[21] Faul F , Erdfelder E , Lang AG , Buchner A . G*power 3: a flexible statistical power analysis program for the social, behavioral, and biomedical sciences. Behav Res Methods. 2007;39(2):175‐191. doi:10.3758/BF03193146 17695343

[22] Serdar CC , Cihan M , Yücel D , Serdar MA . Sample size, power and effect size revisited: simplified and practical approachin pre‐clinical, clinical and laboratory studies. Biochem Med. 2021;31(1):1‐27. doi:10.11613/BM.2021.010502 PMC7745163 33380887

[23] Hu R , Xia CQ , Butfiloski E , Clare‐Salzler M . Effect of high glucose on cytokine production by human peripheral blood immune cells and type I interferon signaling in monocytes: implications for the role of hyperglycemia in the diabetes inflammatory process and host defense against infection. Clin Immunol. 2018;195:139‐148. doi:10.1016/j.clim.2018.06.003 29894743 PMC6119493

[24] Aras M , Tchang BG , Pape J . Obesity and diabetes. Nurs Clin North Am. 2021;56(4):527‐541. doi:10.1016/j.cnur.2021.07.008 34749892

[25] Klein S , Gastaldelli A , Yki‐Järvinen H , Scherer PE . Why does obesity cause diabetes? Cell Metab. 2022;34(1):11‐20. doi:10.1016/j.cmet.2021.12.012 34986330 PMC8740746

[26] Katsogiannos P , Kamble PG , Pereira MJ , et al. Changes in circulating cytokines and Adipokines after RYGB in patients with and without type 2 diabetes. Obesity. 2021;29(3):535‐542. doi:10.1002/OBY.23093 33624436 PMC7986425

[27] Feldman EL , Callaghan BC , Pop‐Busui R , et al. Diabetic neuropathy. Nat Rev Dis Prim. 2019;5(1):41. doi:10.1038/s41572-019-0092-1 31197153

[28] Zheng H , Sun W , Zhang Q , et al. Proinflammatory cytokines predict the incidence of diabetic peripheral neuropathy over 5 years in Chinese type 2 diabetes patients: a prospective cohort study. EClinicalMedicine. 2021;31:100649. doi:10.1016/j.eclinm.2020.100649 33385123 PMC7772538

[29] Choi J , Choi JY , Lee SA , et al. Association between family history of diabetes and clusters of adherence to healthy behaviors: cross‐sectional results from the health examinees‐gem (HEXA‐G) study. BMJ Open. 2019;9(6):e025477. doi:10.1136/BMJOPEN-2018-025477 PMC6588964 31209083

[30] Tsenkova VK , Karlamangla AS , Ryff CD . Parental history of diabetes, positive affect, and diabetes risk in adults: findings from MIDUS. Ann Behav Med. 2016;50(6):836‐843. doi:10.1007/s12160-016-9810-z 27287937 PMC5127745

[31] Zhang X , Zhu Y , Zhou Y , Fei B . Interleukin 37 (IL‐37) reduces high glucose‐induced inflammation, oxidative stress, and apoptosis of podocytes by inhibiting the STAT3‐cyclophilin a (CypA) signaling pathway. Med Sci Monit. 2020;26:e922979‐e922971. doi:10.12659/MSM.922979 32931486 PMC7518013

[32] Ballak DB , Li S , Cavalli G , et al. Interleukin‐37 treatment of mice with metabolic syndrome improves insulin sensitivity and reduces pro‐inflammatory cytokine production in adipose tissue. J Biol Chem. 2018;293(37):14224‐14236. doi:10.1074/jbc.RA118.003698 30006351 PMC6139546

[33] Catalan‐Dibene J , McIntyre LL , Zlotnik A . Interleukin 30 to Interleukin 40. J Interferon Cytokine Res. 2018;38(10):423‐439. doi:10.1089/jir.2018.0089 30328794 PMC6206549

[34] Bastian D , Sui X , Nguyen HD , et al. Interleukin‐23 receptor signaling by interleukin‐39 potentiates T cell pathogenicity in acute graft‐versus‐host disease. Am J Transplant. 2021;21(11):3538‐3549. doi:10.1111/ajt.16624 33934505

[35] Weng L , Huang G , Gong L , et al. Low levels of serum IL‐39 are associated with autoimmune thyroid disease. J Clin Lab Anal. 2022;36(4):e24284. doi:10.1002/jcla.24284 35182078 PMC8993603

[36] Wegner A , Benson S , Rebernik L , et al. Sex differences in the pro‐inflammatory cytokine response to endotoxin unfold in vivo but not ex vivo in healthy humans. Innate Immun. 2017;23(5):432‐439. doi:10.1177/1753425917707026 28443392

[37] Harding AT , Heaton NS . The impact of estrogens and their receptors on immunity and inflammation during infection. Cancers. 2022;14(4):909. doi:10.3390/cancers14040909 35205657 PMC8870346

[38] Roden M , Shulman GI . The integrative biology of type 2 diabetes. Nature. 2019;576(7785):51‐60. doi:10.1038/s41586-019-1797-8 31802013

[39] Khanna D , Khanna S , Khanna P , Kahar P , Patel BM . Obesity: a chronic low‐grade inflammation and its markers. Cureus. 2022;14(2):e22711. doi:10.7759/cureus.22711 35386146 PMC8967417

